# ACE2 Is Expressed in Immune Cells That Infiltrate the Placenta in Infection-Associated Preterm Birth

**DOI:** 10.3390/cells10071724

**Published:** 2021-07-08

**Authors:** Phetcharawan Lye, Caroline E. Dunk, Jianhong Zhang, Yanxing Wei, Jittanan Nakpu, Hirotaka Hamada, Guinever E. Imperio, Enrrico Bloise, Stephen G. Matthews, Stephen J. Lye

**Affiliations:** 1 Department of Physiology, Temerty Faculty of Medicine, University of Toronto, Toronto, ON M5S 1A8, Canada; Tlye@lunenfeld.ca (P.L.); h-hamada@tohoku.ac.jp (H.H.); ebloise@icb.ufmg.br (E.B.); stephen.matthews@utoronto.ca (S.G.M.); 2 Lunenfeld-Tanenbaum Research Institute, Sinai Health, Toronto, ON M5G 1X5, Canada; caroline.dunk@uhnresearch.ca (C.E.D.); jianhong@lunenfeld.ca (J.Z.); Yanxing@lunenfeld.ca (Y.W.); jnakpu@uwo.ca (J.N.); guinever.imperio@mail.utoronto.ca (G.E.I.); 3 Toronto General Hospital Research Institute, University Health Network, Toronto, ON M5G 2C4, Canada; 4 Department of Morphology, Federal University of Minas Gerais, Belo Horizonte 31270-910, Brazil; 5 Department of Obstetrics and Gynecology, Temerty Faculty of Medicine, University of Toronto, Toronto, ON M5S 1A8, Canada; 6 Department of Medicine, Temerty Faculty of Medicine, University of Toronto, Toronto, ON M5S 3H2, Canada

**Keywords:** SARS-CoV-2, COVID-19, ACE2, chorioamnionitis, preterm birth, M1, M2 macrophages, monocyte, neutrophil, lipopolysaccharide (LPS), placenta

## Abstract

COVID-19 is associated with increased incidence of preterm birth (PTB). We assessed pathways by which SARS-CoV-2 could access the placenta. Placentae, from PTB with or without chorioamnionitis (ChA), or from term pregnancies (*n* = 12/13/group) were collected. Peripheral blood was collected from healthy pregnant women (*n* = 6). Second trimester placental explants (16–20 weeks, *n* = 5/group) were treated with lipopolysaccharide (LPS, to mimic bacterial infection) and ACE2, CCL2, IL-6/8 and TNFα mRNA was assessed. ChA-placentae exhibited increased *ACE2* and *CCL2* mRNA expression (*p* < 0.05). LPS increased cytokine and *ACE2* mRNA in placental explants. Placental ACE2 protein localized to syncytiotrophoblast, fetal endothelium, extravillous trophoblast and in immune cells-subsets (M1/M2 macrophage and neutrophils) within the villous stroma. Significantly increased numbers of M1 macrophage and neutrophils were present in the ChA-placenta (*p* < 0.001). Subsets of peripheral immune cells from pregnant women express the ACE2 mRNA and protein. A greater fraction of granulocytes was positive for ACE2 protein expression compared to lymphocytes or monocytes. These data suggest that in pregnancies complicated by ChA, ACE2 positive immune cells in the maternal circulation have the potential to traffic SARS-CoV-2 virus to the placenta and increase the risk of vertical transmission to the placenta/fetus.

## 1. Introduction

A novel coronavirus SARS-CoV-2 (2019-nCoV) appeared in Wuhan, China on December 30, 2019, and spread rapidly around the world [1]. At present, it is described that mother to fetus/neonate vertical transmission of COVID-19 infection occurs in <5% of all COVID-19 positive pregnancies [2]. In this context, we [3] and others [4] have suggested that this may result from a low expression of the SARS-CoV-2 cell entry protein angiotensin-converting enzyme 2 (ACE2) on placental trophoblast cells in mid-late pregnancy, even though ACE2 immunoreactivity is found in first, second and third-trimester placentae [5]. ACE2 is an active peptide of the renin–angiotensin system (RAS) which has been given considerable attention as a potential target for anti-viral therapeutics [6], since it is the main cell entry receptor of SARS-CoV-2 [7]. Other SARS-CoV-2 cell entry proteins have been described [8]. However, ACE2 is predominantly expressed in the syncytiotrophoblast layer (which directly faces the maternal blood), compared to other SARS-CoV-2 cell entry proteins [9].

A systematic review meta-analysis of 13 publications related to COVID infection in pregnancies from China initially suggested a high rate of maternal and neonatal complications in COVID-19 infected individuals, with a preterm birth rate of 20% and a lower neonatal infection rate of 6% [10]. Recent publications following the spread of COVID 19 around the world have further elucidated the risk of pregnancy complications associated with COVID 19 infection dependent on time of infection in pregnancy; reporting that the rate of PTB is higher in women who are infected after 33 weeks and is primarily due to iatrogenic PTB/delivery due to worsening maternal conditions and not an increase in spontaneous PTB [11,12]. Despite the fact that most cases of PTB can be iatrogenic, PTB in COVID-19-positive pregnant women is still very high and may range up to 63.8% [13,14,15,16], providing impetus to define the mechanisms and clinical conditions in which COVID 19 may be associated with PTB. Furthermore, COVID-19 can possibly induce fetal demise [17], since 5 fetal deaths associated with acute chorioamnionitis from women pregnant at 21–38 weeks of gestation experiencing a COVID 19 infection (but bearing no comorbidities or pregnancy complications and not taking any medication) have been reported. More interestingly, pathologic findings from placentas of pregnant women with COVID-19 have been variable—some reports describe no significant changes, some describe evidence of either fetal or maternal vascular malperfusion or both, and others describe inflammatory lesions including chronic histiocytic intervillositis, villitis, funisitis, and chorioamnionitis, most coming from non-infected placentas [18,19,20,21,22,23,24]. However, there is now increasing data showing that in infected maternal fetal dyads where SARS-CoV-2 placental infection is confirmed by PCR, immunohistochemistry or in situ hybridisation, the pathology is more severe and includes trophoblast necrosis, acute villitis and intervillositis and increased fibrin deposition [17,25,26].

The immune system is a core component of the maternal-fetal interface. Disruption of the immune system of a pregnant woman, as in the case of bacterial infection, places the mother and fetus/neonate at risk of other infections, such as viruses, which can impact the course of pregnancy for both mother (e.g., preterm birth) [27] and fetus/neonate (e.g., systemic inflammatory response syndrome) [28]. Additionally, it has been suggested that viral infection of the placenta modifies the immune response to bacterial products by destroying the normal ‘tolerance’ to lipopolysaccharide (LPS) and aggravates the inflammatory response, which in turn leads to preterm labor [29,30]. Immune cells at the maternal-fetal interface play a critical role in a successful pregnancy. In the first trimester, they contribute to remodeling of the uteroplacental circulation [31], and at term an influx of maternal peripheral monocytes into the decidua and myometrium leads to their differentiation into macrophages which generate inflammatory mediators contributing to the initiation of labor [32,33]. However, these cells also contribute to pathologies of pregnancy. For example, in cases of chorioamnionitis, bacterial infection of the fetal membranes and/or placenta results in an increased influx of maternal neutrophils and also monocytes which subsequently differentiate into classical activated inflammatory macrophage [34,35]. The potential adverse consequences to the fetus of placental infection and sensitization to maternal-fetal viral transmission and the mechanisms by which this might occur require further investigation.

The syncytiotrophoblast (STB) provides the interface between the maternal blood, containing peripheral immune cells, and the extraembryonic tissues and fetus [19,36,37]. Interestingly, activated macrophages in patients with heart failure have been shown to express high levels of the SARS-CoV-2 receptor, ACE2 [38]. Furthermore, in patients with chronic obstructive pulmonary disease (COPD) activation of neutrophils, NK cells, Th17 cells, Th2 cells, Th1 cells, dendritic cells, macrophage and TNFα secreting cells can be induced by overexpression of ACE2 leading to a severe inflammatory response [39]. Since these immune cells can potentially engage the SARS-CoV-2 virus, they may also represent a reservoir for the virus and a vector for the virus to infect the placenta/fetal membranes.

We hypothesized that the presence of an intrauterine bacterial infection will activate peripheral maternal monocytes and neutrophils and cause them to target the uterus and intrauterine tissues. If these immune cells express ACE2, then these cells would have the potential to transport virus to the placenta in cases of maternal SARS-CoV-2 infection and thereby increase the risk of placental infection and potential vertical transmission of the virus to the fetus.

## 2. Materials and Methods

In our study, we included pregnancies (25.3–36.0 weeks) complicated by preterm birth (PTB; spontaneous PTB of unknown causes (*n* = 9), PPROM (*n* = 1), vaginal bleeding from known placenta previa (*n* = 1), emergency C-Section (*n* = 1); total *n* = 12) or preterm birth with chorioamnionitis (ChA; unknown causes (*n* = 8), group B streptococcus (GBS) (*n* = 1), acute ChA (*n* = 2), ChA with fetal inflammatory response (*n* = 2); total *n* = 13, identified as acute histologic grade 1–3 by placental pathologist) or from term pregnancies (>37 weeks’ gestation) from healthy women following spontaneous term labour (vaginal delivery, SVD; *n* = 13) or not in labour (elective caesarean section, ELCS; *n* = 13) (Table 1) [40] (further details are provided in Table 1).

Second trimester placental tissue was collected at 16–20 weeks of pregnancy (*n* = 5/group) from elective pregnancy terminations. Peripheral blood was collected from normal healthy pregnancies (*n* = 6). Exclusion criteria included pregnancies exhibiting respiratory and cardiovascular conditions, cervical incompetence, diabetes, fetal growth restriction, fetal malformation, hypertension, multiple gestation, preeclampsia, sexually transmitted diseases, thyroid disease and uterine malformations [40,41]. Biospecimens were collected by the Research Centre for Women’s and Infants’ Health (RCWIH) BioBank staff at Mount Sinai Hospital (Toronto, ON, Canada) following informed written consent (process n# 20–0101-E) and procedures approved by Sinai Health System and the University of Toronto Research Ethics Board. 

### 2.1. Placental Explant Culture 

Placental explant cultures were conducted as previously described [42]. Tissues were dissected into villous clusters (~15–30 mg), and cultured in DMEM with 1% insulin, transferrin, and selenium-A (Invitrogen, Grand Island, NY, USA) equilibrated in 8% O_2_–5% CO_2_, at 37 °C). Explants were treated with LPS from *Escherichia coli* (10 μg/mL L4391, Sigma-Aldrich, St. Louis, MO, USA) or vehicle for 4 or 24 h [42] before storage at −80 °C.

### 2.2. Immunohistochemistry

Placental tissues (*n* = 6/group) were processed for immunohistochemical analysis as previously described [43]. Tissue sections (5 um) were deparaffinized, rehydrated, and subjected to heat mediated antigen retrieval. After protein blocking (Dako, Mississauga, ON, Canada), slides were incubated overnight (4 °C) with primary antibodies: anti-rabbit ACE2 (1:200, ab15348, Abcam, Toronto, ON, Canada) rabbit anti-Human MHC-II (M1 marker) (1:400, ab180779, Abcam), anti-rabbit mannose receptor (M2 marker) (CD206, 1:200, ab64693, Abcam), anti-mouse CD68 (macrophage) (1:100, Dako) and anti-rabbit neutrophil elastase (NE, 1:100, ab21595, Abcam). After incubation, the slides were washed and incubated with the corresponding biotinylated secondary antibody (1:300, 1 h, Dako), washed and incubated with streptavidin-HRP (Dako); immunostaining was detected with the peroxidase substrate kit DAB (Dako). Slides were counterstained with hematoxylin. Negative controls were performed using either isotype Mouse IgG1 or rabbit IgG/IgG1 antibodies.

### 2.3. Image Analysis and Quantification

Quantification of positive-stained placental macrophage subtypes and neutrophils (*n* = 6/group) was performed using newCAST software (Visopharm, Boulder, CO, USA), as previously described [44], using a standard protocol that assigned random counting frames covering 5% of each total masked tissue area. A positively-stained ratio was generated by dividing the total numbers of brown, positively-stained cells by the total number of cells in the tissue area).

### 2.4. Immunofluorescence Staining

ChA placental tissue slides were deparaffinized, rehydrated and subjected to heat mediated antigen retrieval. Following procedures to reduce autofluorescence and non-specific binding, placental slides were incubated with primary antibodies anti-rabbit ACE2 (1:100, ab15348, Abcam), anti-mouse CD68 (1:100, Dako), anti-mouse IgG1 (Dako) and anti-rabbit IgG1 (ab171870, Abcam) added as an isotype control overnight (4 °C). Slides were washed and incubated with fluorescent secondary antibodies, using either the anti-mouse Alexa 488 (1:1000) or the anti-rabbit Alexa 594 (1:1000) secondary antibodies (Thermo Fisher Scientific, Mississauga, ON, Canada) and counterstained with DAPI. Fluorescent microscopy was performed using a spinning disc confocal microscope at various magnifications.

To localize ACE2 + MHC-II (M1), ACE2 + CD206 (M2) and ACE2 + Neutrophil elastase (NE) (primary antibodies from same species), immunofluorescence experiments were performed as described previously [45,46]. Placental sections from ChA pregnancies were deparaffinized, rehydrated, and subjected to antigen retrieval. Following procedures to reduce autofluorescence and non-specific binding, placental sections were incubated overnight with primary antibodies ACE2 (1:100, ab15348, Abcam). Slides were washed and incubated with secondary antibodies using anti-rabbit Alexa 594 (1:1000) and subsequently washed, blocked and incubated with primary antibodies anti-rabbit MHC II (M1) (1:100, Abcam), anti-rabbit mannose receptor (CD206, M2,1:100, Abcam) anti-rabbit NE (1:100, Abcam) and anti-rabbit IgG1 (isotype control) overnight (4 C). Sections were incubated with the corresponding biotinylated secondary antibody (1:300, 1 h, Dako), washed, incubated with streptavidin-Alexa488 and nuclei stained with DAPI. Fluorescent microscopy was performed using a spinning disc confocal microscope at various magnifications.

### 2.5. Quantitative Real Time PCR (qPCR)

*ACE2*, interleukin *(IL)-6*, *IL-8*, tumor necrosis factor *(TNF)-α* and chemokine ligand 2 *(CCL2)* mRNA levels were measured by qPCR as previously described [3] and expression normalized to the geometric mean of selected reference genes, including DNA topoisomerase 1 *(TOP1)*, 14-3-3 protein zeta/delta *(YWHAZ)* and TATA-box binding protein *(TBP)*. The relative expression of target genes was calculated by the 2^−ΔΔCT^ method [47]. The primer sequences of all the assessed genes are shown in Table 2.

### 2.6. Immunoblotting

Western blot analysis was conducted as previously described [43] using anti-rabbit ACE2 (dilution 1:1000; Abcam, ab108209) and anti-goat β-actin (dilution 1:2000; Santa Cruz Biotechnology, Dallas, TX, USA).

### 2.7. Immune Cell Isolation 

Monocytes and lymphocytes were isolated separately from peripheral blood samples from healthy pregnancies using the RosetteSep system (Stemcell Technologies, Vancouver, BC, Canada) as previously described [49]. Primary human neutrophils were isolated by Histopaque double density gradient method, (Sigma-Aldrich) as previously described [50].

### 2.8. Flow Cytometry

Whole blood (50 µL) was stained with LIVE/DEAD^®^ fixable cell stain kit (L/D-violet; Invitrogen) and then incubated (30 min) with human Fc block (BD Pharmingen, Franklin lakes, NJ, USA). ACE2 surface staining was conducted by Alexa Fluor^®^ 700-conjugated mouse anti-human ACE-2 (R&D Systems, Minneapolis, MN, USA) and APC-H7 mouse anti-human CD45 (BD Biosciences) antibodies. Data were acquired with a Gallios flow cytometer (Beckman Coulter, Pasadena, CA, USA) and analyzed using FlowJo V10 (TreeStar) or Kaluza 2 (Beckman Coulter) software. Circulating lymphocytes, monocytes and granulocytes were recognized by their distinct morphological features in forward and side scatter distribution.

### 2.9. Statistical Analysis

Data analyses were performed with Prism v8 (GraphPad Software, San Diego, CA, USA); qPCR data were assessed for normal distribution using D’Agostino and Pearson or the Shapiro-Wilk test; outliers were identified using “QuickCalcs” (GraphPad Software). All data were normally distributed with the exception of the flow cytometry. Gene and protein expression, number of macrophages, and maternal immune cells were analyzed using one-way ANOVA followed by Tukey’s multiple comparisons test. Results from LPS-treated explants were assessed by paired t-test. For flow cytometry statistical analyses were performed by R software (3.4.3). Multiple comparisons were conducted by Kruskal-Wallis test and Wilcoxon test was followed to examine the statistical difference between two groups. Significance was set at *p* < 0.05. 

## 3. Results

### 3.1. Chorioamnionitis and LPS Exposure Are Associated with Increased Placental ACE2 Expression and an Inflammatory Cytokine/Chemokine Response

**Figure 1:**
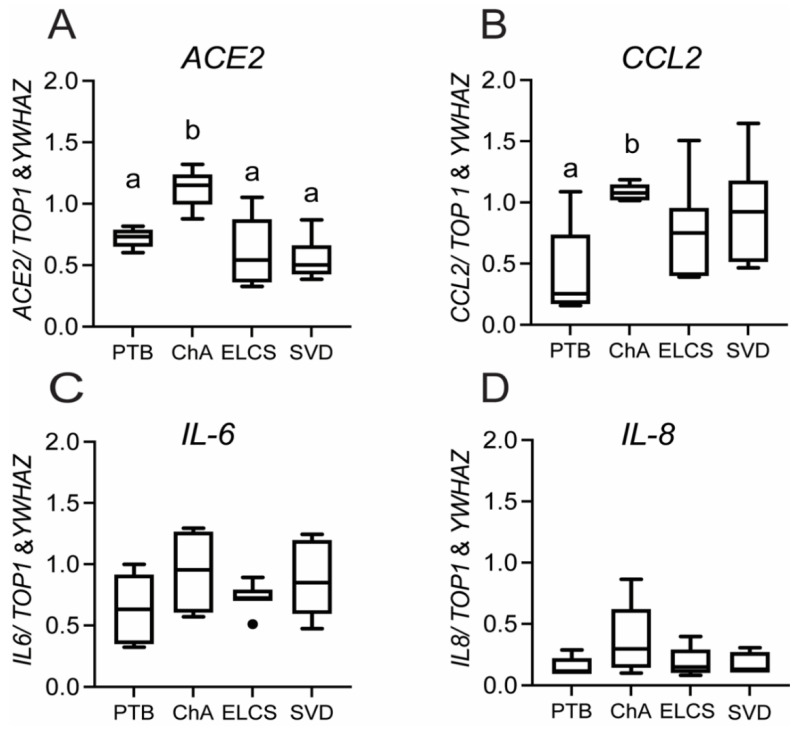
*ACE*, and chemokine mRNA expression is increased in placenta of pregnancies complicated by preterm labor with chorioamnionitis (ChA). Bar charts show the relative levels of *ACE2* (**A**), *CCL2* (**B**), *IL-6* (**C**), and *IL-8* (**D**) mRNA in placentas from PTB, ChA, SVD and ELCS deliveries, assessed by real time qPCR. Data are normalized by the geometric mean of *TOP1* and *YWHAZ* (reference genes), *n* = 6/7/group. Statistical differences were tested by Tukey’s multiple comparisons test. Data are presented as mean ± SEM. Different letters indicate a difference between groups of *p* < 0.05.

Placental *ACE2* mRNA expression was increased (*p* < 0.01) in preterm pregnancies complicated with ChA compared with PTB alone, SVD or ELCS (at term) (Figure 1A).

**Figure 2:**
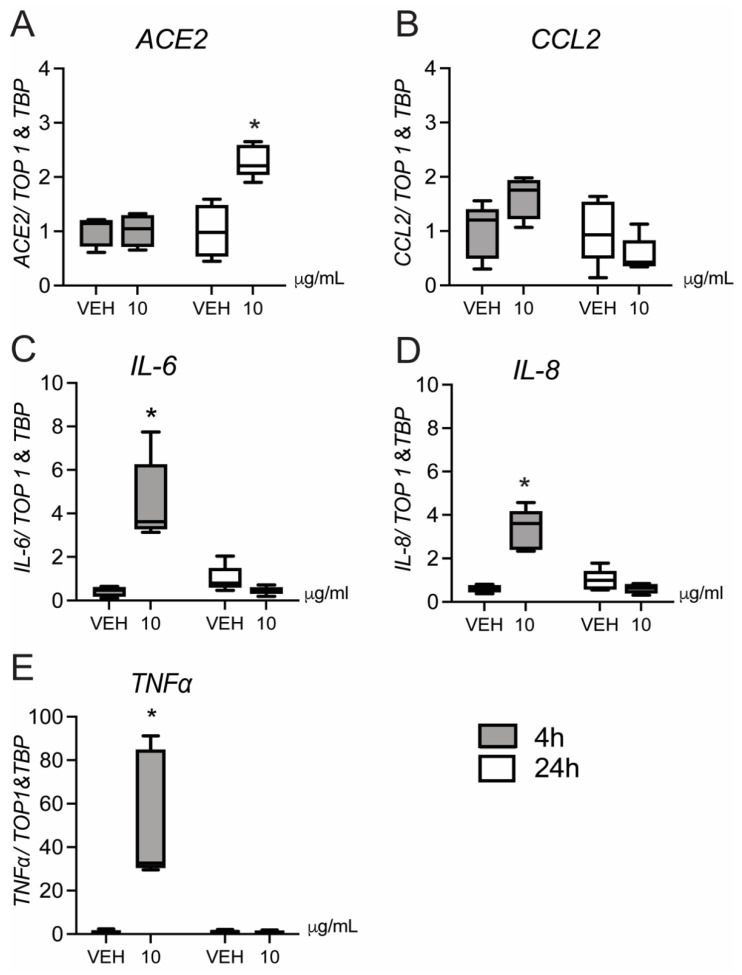
Effect of LPS on *ACE2*, *CCL2*, *IL-6/8* and *TNFα* mRNA expression in second trimester human placental explants. Second trimester placental explants were treated with LPS (10 µg/mL), or vehicle for 4 and 24 h and mRNA levels of *ACE2* (**A**), *CCL2* (**B**), *IL-6* (**C**), *IL-8* (**D**) and *TNFα* (**E**), were quantified by qPCR (*n* = 5/group). Data are normalized by the geometric mean of *TOP1* and *TBP* (reference genes). Data are expressed as means ± SEM. Statistical differences were tested using a paired *t*-test. * *p* < 0.05, versus vehicle.

*CCL2* (but not *IL6/8*) mRNA levels were also increased in ChA (Figure 1B; *p* < 0.05) compared to gestational-age matched patients without ChA. No significant change in the expression of IL6/8 was detected in any group (Figure 1C,D). Treatment of 2nd trimester (a time when PTB has greater adverse effects on neonatal outcome) placental explants with LPS induced a rapid (4 h) increase in expression of *IL-6/8* and *TNFα* mRNA (*p* < 0.05; Figure 2D,E), as well as a strong trend (*p* = 0.06) towards higher expression of the chemokine *CCL2* (Figure 2B). 

Cytokine/chemokine expression returned to basal levels 24 h post-LPS treatment, at which time expression of *ACE2* mRNA was increased (*p* < 0.05) (Figure 2A). This time course suggests that ACE2 expression may be induced by the early increase in cytokines.

**Figure 3:**
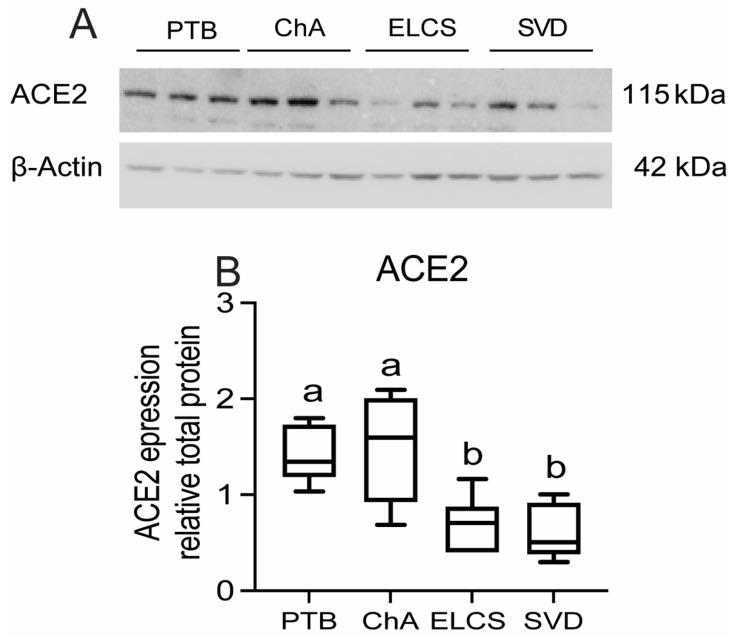
Placental ACE2 protein levels are increased in PTB with/without chorioamnionitis (ChA) as compared to term. Protein lysates of placentas from PTB, ChA, ELCS and SVD deliveries were assessed for level of ACE2 protein by western blotting. (**A**) Representative western blot images and (**B**) densitometric analysis of ACE2 protein level, normalized by *β*-actin (loading control for protein), *n* = 6/group. Statistical differences were tested by Tukey’s multiple comparisons test. Data are presented as mean ± SEM. Different letters indicate a difference between groups of *p* < 0.05.

In contrast to the elevated expression of *ACE2* mRNA, we did not detect any increase in ACE2 total protein within the placentas of ChA pregnancies, when compared to that of age-matched PTB, although the levels of ACE2 protein in preterm placentas (with or without ChA) were higher (*p* < 0.05) than in placentas at term (Figure 3A,B).

**Figure 4:**
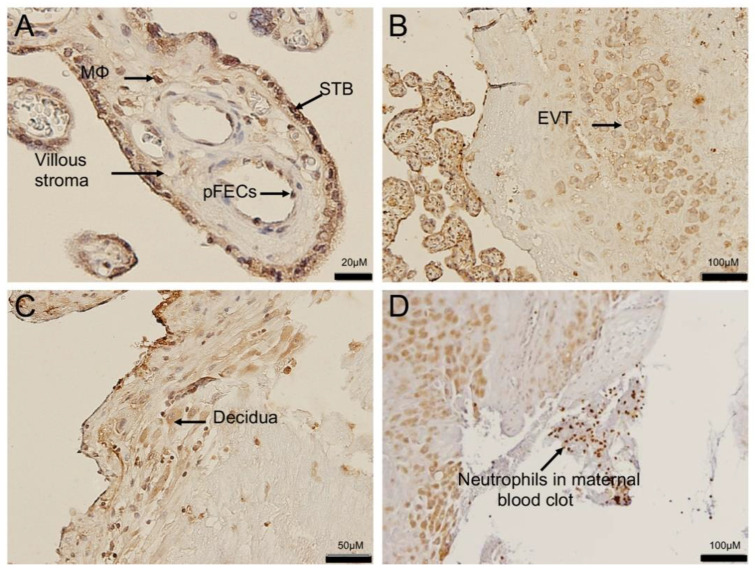
ACE2 localization in the placenta and in maternal intervillous immune cells. Representative images of ACE2 immunostaining in a placenta from an uncomplicated PTB delivery (**A**) ACE2 staining was localized predominantly in the syncytiotrophoblast layer (STB), fetal blood vessel (pFECs) and fetal hoefbauer cells (MΦ) of the placental villi. (**B**) ACE2 also localized to extravillous trophoblast (EVT) within the basal plate of the placenta and in (**C**) decidual stromal cells. (**D**) ACE2 expression in maternal neutrophils of maternal blood clot adjacent to the decidua, Sections were counter-stained with hematoxylin. *n* = 6/group.

Since ACE2 total protein was not increased with ChA, we investigated its localization in specific cellular compartments in these placentas. ACE2 was expressed in the syncytiotrophoblast (STB), and endothelium of fetal capillaries, sites of contact with maternal and fetal blood, respectively. ACE2 immunoreactivity was also present in macrophage within the placental villous stroma and in fetal endothelial cells (Figure 4A).

ACE2 expression was also present in extravillous trophoblast (EVT) (Figure 4B) and in decidual stromal cells within the placental basal plate (Figure 4C), as well as immune cells within maternal blood present in the intervillous space (Figure 4D).

**Figure 5:**
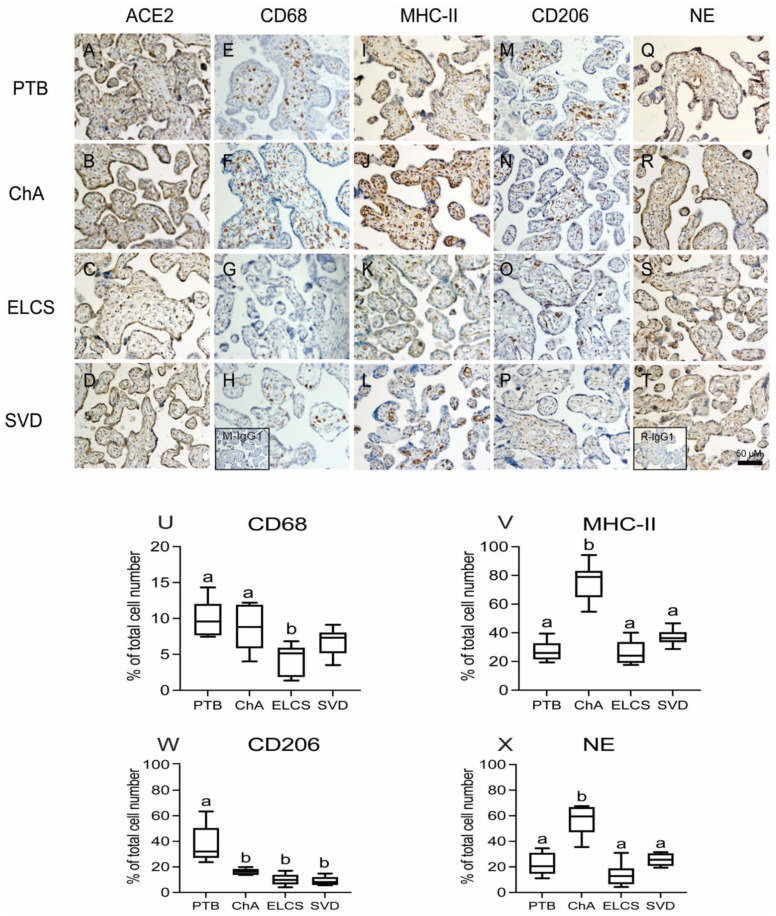
Localization and quantitation of placental ACE2, CD68 (macrophage marker), MHC-II (M1 marker), CD206 (M2 marker) and neutrophil (NE) staining in complicated and uncomplicated pregnancies. Representative images of ACE2 (**A**–**D**), M1 (MHCII **E**–**H**), M2 (CD206 **I**–**L**), CD68 (**M**–**P**), and NE (**Q**–**T**) staining in preterm and term placentas (*n* = 6 in each group). Inserts (bottom left) in H mouse IgG1 and T rabbit IgG1 isotype control staining. Scale bar represents 50 μm. Image analysis quantitation of positively stained immune cells as a proportion of the total cells number was performed on a randomly selected 5% of the total tissue area of each placental section. (**U**) CD68 (**V**) M1 (**W**) M2 (**X**) NE. Statistical differences were tested by Tukey’s multiple comparisons test. Data are presented as mean ± SEM. Different letters indicate a difference between groups of *p* < 0.05.

The finding of ACE2 expression in histologically-identified immune cells within the placenta led us to undertake further characterization of these cells, using monoclonal antibodies specific for the macrophage lineages (CD68), M1 macrophage (MHC-II), M2 macrophage (CD206) and neutrophils (neutrophil elastase; NE). ACE2 was localized to syncytiotrophoblast of all pregnancy groups (Figure 5A–D) with reduced staining in term compared to preterm groups. Macrophages were also identified within the placental villi of all pregnancy groups (Figure 5E–H) and further analysis identified these to be both M1 (inflammatory) and M2 (angiogenic) sub-types. (Figure 5I–P). The M1 and M2 macrophages were present throughout the stroma as well as associated with fetal blood vessels. Staining with NE detected the presence of neutrophils within the placental vasculature and villi of all pregnancy groups (Figure 5Q–T). Total macrophage (CD68+) numbers were similar in ChA and PTB groups but both had higher numbers than the term groups (SVD, ELCS; (*p* < 0.01) (Figure 5U). 

Importantly, the number of MHC-II+ M1 (inflammatory) macrophage was increased (*p* < 0.001) specifically in ChA patients compared to PTB, SVD and ELCS groups (Figure 5V), whereas, CD206+ M2 (angiogenic) macrophage were increased (*p* < 0.001) in PTB patients compared to ChA, SVD and ELCS groups (Figure 5W). Neutrophil numbers were also higher in ChA pregnancies than in the PTB, SVD or ELCS groups (Figure 5X; *p* < 0.001).

**Figure 6:**
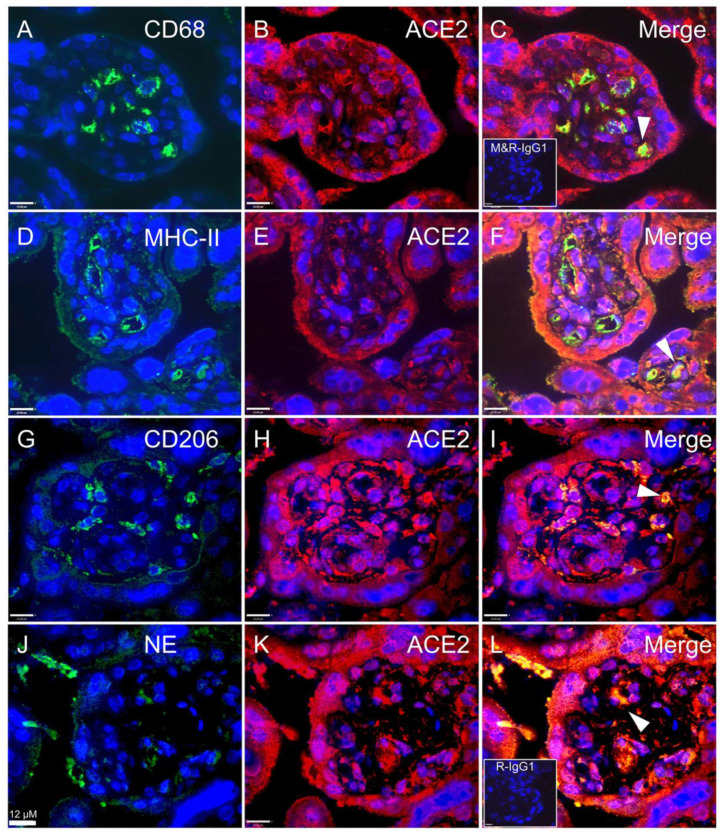
Co-localization of SARS-CoV-2 associated cell-entry protein, ACE2, within specific immune cell populations in ChA placenta. Representative images of ACE2, CD68, M1, M2 and NE staining in immunofluorescence (ACE2; red color and M1, M2, macrophage and NE; green color), macrophage co-staining with ACE2 (**A**–**C**) M1 co-staining with ACE2 (**D**–**F**), M2 co-staining with ACE2 (**G**–**I**) and neutrophil co-staining with ACE2 (**J**–**L**). Co-localization of ACE2 and markers of macrophage and neutrophil confirmed the ACE2 localization within placental macrophages and neutrophils. ACE2 expressing immune cells were present in the villous stroma and adjacent to fetal blood vessels. Only a subset of each of immune cells expressed ACE2. Arrows show ACE2 staining within CD68, M1, M2 and NE stained cells. Sections were counter-stained with DAPI (blue color) or co-staining (yellow color). Inserts (bottom left) in (**C**) mouse and rabbit IgG1 isotype control and (**L**) rabbit IgG1 isotype control staining. *n* = 3/group. Scale bar represents 12 μm.

Next, we determined whether ACE2 is localized within infiltrated immune cell populations in the ChA placenta, by undertaking co-localization immunofluorescence analysis of ACE2 with CD68 (macrophage), MHC-II (M1), CD206 (M2) and NE (neutrophil) markers. ACE2 was expressed in macrophages (M1 and M2) and neutrophils, confirming our histological findings. Immune cells expressing ACE2 were localized in the villous stroma and adjacent to fetal blood vessels (Figure 6).

### 3.2. Expression of ACE2 in Circulating Maternal Immune Cells

**Figure 7:**
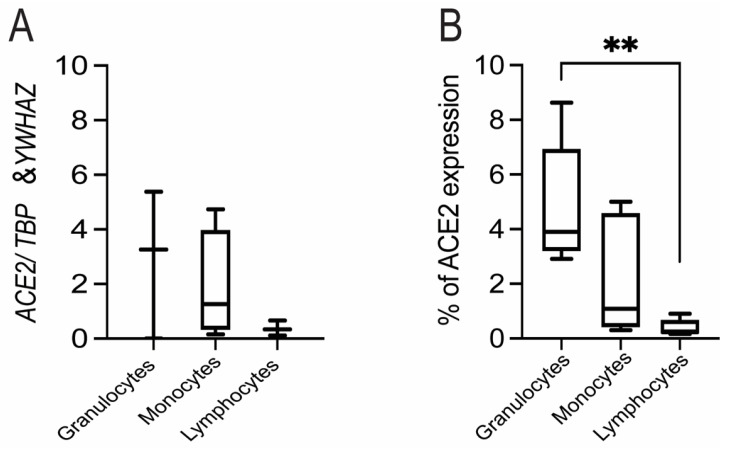
The surface expression of ACE2/*ACE2* on circulating granulocytes, monocytes and lymphocytes in pregnant women. (**A**) The relative expression of *ACE2* mRNA on granulocytes, monocytes and lymphocytes in maternal peripheral blood. There were no differences between groups (*n* = 4/group) as tested by Tukey’s multiple comparisons test. Data are presented as mean +/-SEM. (**B**) Peripheral human blood cells were stained with anti-human ACE2 and CD45 antibodies. The percentage of ACE2+ cells was calculated according to the gating of viable CD45+ granulocytes, monocytes and lymphocytes. Statistical analyses were performed by R software (3.4.3). Multiple comparisons were conducted by Kruskal-Wallis test followed by Wilcoxon test to examine the statistical difference between two groups. *n* = 6/group. **, *p* < 0.005.

Expression of *ACE2* mRNA was detected in circulating granulocytes, monocytes and lymphocytes from pregnant women, though levels were quite variable and were not significantly different across cell types (Figure 7A).

Flow cytometry also revealed that ACE2 was expressed in fractions of circulating granulocytes, monocytes and neutrophils. A greater fraction of circulating granulocytes was found to be positive for ACE2 than lymphocytes, but not monocytes (*p* < 0.005; Figure 7B).

## 4. Discussion

We provide evidence that under conditions of intrauterine infection/inflammation there is an influx of ACE2-expressing neutrophils and monocytes (macrophage) into the placenta. We demonstrated that ACE2 mRNA levels were increased in the placenta of pregnancies complicated by chorioamnionitis compared to placentas from patients of similar gestational age with no evidence of infection, or from term pregnancies (collected from non-laboring or laboring deliveries). Despite this increase in mRNA, placental ACE2 total protein levels were not different in patients with chorioamnionitis compared to PTB alone, although both preterm groups expressed higher levels of ACE2 than placentas from patients at term (from laboring vaginal or non-laboring elective caesarian deliveries). This led us to investigate the expression of ACE2 protein in specific cell types within the placenta/decidua. ACE2 was localized to the STB, which is exposed to the maternal circulation, as well as the vascular endothelium of the fetal circulation. In addition, extravillous trophoblast (EVT) within the decidua and decidual stromal cells were positive for ACE2. Importantly, ACE2 was also expressed in immune cells (macrophage and neutrophils) within the placenta of ChA patients, many of which were identified in close proximity to, or within, the wall of fetal blood vessels. Greater numbers of MHC-II+ M1 macrophages and NE+ neutrophils were found in pregnancies complicated by ChA than the other pregnancy groups. In agreement with previous reports, fetal Hofbauer cells expressing the M2 marker CD206 were the predominant macrophage subtype in the non-infected groups. ACE2 mRNA and protein were detected in specific immune subsets (neutrophils, monocytes, lymphocytes) in maternal peripheral blood, with the fractions of neutrophils expressing ACE2 being higher than in lymphocytes (but not monocytes).

LPS treatment of placental explants induced a rapid induction of chemokine/cytokine (CCL2, IL-6/8 and TNFα) transcripts 4 h post exposure, followed by *ACE2* mRNA at 24 h. Since syncytiotrophoblast and placental resident immune cells express ACE2, two potential routes of potential vertical transmission with SARS-CoV2 might exist, one through direct interaction between virus in the peripheral maternal blood that baths the outer (syncytial) surface of the placenta, and one through the influx of virus possibly contained in infiltrating immune cells.

There is conflicting data as to whether SARS-CoV-2 can be transmitted to the fetus through the placenta in women with COVID-19. Early reports of obstetric and neonatal outcomes of pregnant women from China with COVID-19 suggested that SARS-CoV-2 infection in pregnancy results in limited neonatal infection [51,52]. However, an increasing number of studies report neonatal cases with congenital or intrapartum infection [53]. In addition, in one preterm pregnancy case, PCR tests of both amniotic fluid and the infant after 24 h were positive, revealing the early exposure and persistence of vertical transmission [54]. Three other neonates showed increased immunoglobulin M (IgM) antibodies specific to COVID19 at birth [55]. There is also a growing body of evidence demonstrating SARS-CoV-2 infection of the placental membrane and the villous trophoblasts via a variety of different laboratory techniques such as PCR [56,57,58], positive strand in situ hybridization [58], immunohistochemistry [56,57], and transmission electron microscopy [59]. These studies have shown that SARs-CoV-2 infection is limited to the syncytiotrophoblast, and is rare occurring in approximately 2% of all cases [60].

Previous reports found minimal expression of *ACE2* mRNA in the placenta [3,4,61,62]. However, we recently demonstrated that placental expression of *ACE2* mRNA is gestational-age dependent with the highest levels in early pregnancy and low to undetectable levels towards term [3] and other studies showed ACE2 is highly expressed in STB, endothelial/perivascular cells and EVT at the maternal-fetal interface early in pregnancy [63]. Of importance, ACE2 levels may be dysregulated in different obstetric pathologies. In this context, decreased levels of placental *ACE2* mRNA in human fetal growth restriction pregnancies [64] and increased levels of maternal plasma ACE2 in early-mid pregnancy in women delivering small for gestational age babies [65] have been reported. Interestingly a recent report of placentas from 5 COVID infected deliveries reported detection of the SARs-CoV-2 spike protein in all cases and in all of these compartments at the maternal-fetal interface, including trophoblasts, decidual stromal cells, immune cells, epithelial cells of the placenta and amnion and endothelial cells of the umbilical cord [66]. These results support the hypothesis that ACE-2 expression by the fetal maternal tissues does provide a route for SARs-CoV-2 to infect the placenta and decidua.

While maternal and fetal neutrophils are present in the amniotic fluid of women with confirmed intraamniotic infection and/or inflammation [67], we did not determine whether the ACE2 expressing immune cells within the placental villi were of maternal or fetal origin. Nevertheless, the surface expression of ACE2 on peripheral immune cells, provides a route by which the virus could enter these cells in the maternal circulation and pass to the placenta in the intervillous space; as has been suggested to occur in the lung [68]. Although our study is limited by the lack of placental tissues from COVID-19 positive deliveries, here we suggest a model (graphical abstract) in which an intrauterine infection (as in the case of chorioamnionitis) leads to the release of cytokines/chemokines that activate maternal immune cells (particularly neutrophils and monocytes/macrophage) and cause them to target and infiltrate the placenta [69,70]. Since a proportion of these cells express the ACE2 protein it is possible that in women who are infected with the SARS CoV-2 virus, these immune cells could also be infected with the virus, and thus provide a direct path for the transmission of viral particles into the placenta and membranes, and in some cases subsequently to the fetus. A recent study has demonstrated that SARS-CoV-2 can infect monocytes and macrophages derived from monocytes (MDMs) [71]. Additionally, SARS-CoV-2 has also been demonstrated to infect alveolar macrophages, which in turn respond by producing T cell chemoattractants [72]. Thus, we propose that circulating maternal immune cells could act as the vector for SARS CoV-2 to infect the placenta and/or fetus. Moreover, since the activated immune cells infiltrating into the placenta can release proinflammatory cytokines this might further enhance placenta *ACE2* mRNA expression, virus entry and infection of the placental bed, particularly in the cases with intrauterine bacterial infection [69]. Whether bacterial products (LPS) can sensitize the placenta to infections by SARS-CoV-2 is not known and requires further study. However, previous reports found LPS-induced inflammation (48h) increases the number of macrophage in term placental explants [73]. It is relevant to note that acute and chronic chorioamnionitis also increases the risk of vertical transmission of the human immunodeficiency virus-1 [74,75]. Since pregnant women with COVID-19 have higher odds of preterm birth [76], future studies are required to assess whether ACE2 expressing maternal peripheral immune cells infected by SARS-CoV-2 can traffic to the placenta and be involved with increased risk of preterm birth and or placental COVID infection in pregnant women with Covid-19.

## 5. Conclusions

Our data suggest that the presence of an intrauterine bacterial infection results in the infiltration of ACE2 expressing maternal peripheral blood monocytes (M1 macrophage) and neutrophils into and across the placental tissues. These ACE2 expressing immune cells have the potential to transport virus to the placenta in cases of concomitant COVID-19 infection and thereby increase the risk of placental infection, PTB and vertical transmission of the virus to the fetus.

## Figures and Tables

**Table 1:** Clinical profile of placental tissues. BMI, (Body Mass Index); V, (Vaginal), C, (Caesarean section with no labor); CL, (Caesarean section with labor); G1, (Grade1); G2, (Grade2); G3, (Grade3); Y/N, (Yes/No); U (Unknown). Values with different superscript letters were significantly different from other patient groups (*p* < 0.05).

Pregnancies	PTB	ChA	ELCS	SVD	*p* Value	Flow Cytometry
Number of patients	(*n* = 12)	(*n* = 13)	(*n* = 13)	(*n* = 13)		(*n* = 6)
**Maternal Characteristics**						
Maternal Age (years)	29.6 ± 1.83 ^a^ (21–43)	28.5 ± 2.25 ^a^ (17–40)	35.8 ± 0.85 ^b^ (34–42)	32.4 ± 1.06 ^a^ (27–43)	<0.05	35.0 ± 3.52 (29–39)
Labor (Y/N/U)	10:2:0	12:1:0	0:13:0	13:0:0	-	0:6:0
BMI	22.4 ± 1.18 ^a^ (18.26–29.75)	23.8 ± 1.12 ^a^ (18.30–29.75)	22.6 ± 1.77 ^a^ (15.24–32.44)	23.2 ± 1.34 ^a^ (19.08–32.91)	NS	23.4 ± 2.91 (19.9 ± 7.3)
**Fetal Characteristics**						
Gestational age (weeks)	31.7 ± 0.93 ^a^ (25.3–36.0)	29.3 ± 1.01 ^a^ (26–33)	38.6 ± 0.30 ^b^ (37–41)	39.0 ± 0.32 ^b^ (37–40.4)	<0.05	39.2 ± 0.08 (39.1–39.3)
Blood draw: Gestational age (weeks)	-	-	-	-	-	14.4 ± 3.36 (12.3–20.9)
Neonatal sex (Male/Female/U)	9:3:0	9:4:0	8:5:0	7:6:0	-	3:0:3
**Pathology Characteristics**						
Chorioamnionitis (G1,G2,G3,U)	No	1:10:1:U	No	No	-	No
Glucocorticoid treatment	Yes	Yes	No	No	-	No

**Table 2:** List of primers used in this study.

Primer Name	Sequence	Reference
*ACE2*	Forward: 5′-GGAGTGATAGTGGTTGGCATTGTC-3′	*
	Reverse: 5′-GCTAATATCGATGGAGGCATAAGGA-3′	
*IL-6*	Forward: 5′-TGCAGAAAAAGGCAAAGAAT-3′	[43]
	Reverse: 5′-CTGACCAGAAGAAGGAATGC-3′	
*IL-8*	Forward: 5′-TGGGAACAAGAGGGCATCTG-3′	[43]
	Reverse: 5′-CCACCACTGCATCAAATTCATG-3′	
*CCL2*	Forward: 5′-TTCATTCCCCAAGGGCTCGCTCA-3′	[42]
	Reverse: 5′-AGCACAGATCTCCTTGGCCACAA-3′	
*TNFα*	Forward: 5′-CCTGGGGAACTCTTCCCTCTGGGG-3′	*
	Reverse: 5′-CAGGCGCCACCACGCTCTTC-3′	
*TBP*	Forward: 5′-TGC ACA GGA GCC AAG AGT GAA-3′	[48]
	Reverse: 5′-CAC ATC ACA GCT CCC CAC CA-3′	
*YWHAZ*	Forward: 5′-CCGCCAGGACAAACCAGTAT-3′	[48]
	Reverse: 5′-CAC ATC ACA GCT CCC CAC CA-3′	
*TOP1*	Forward: 5′-GATGAACCTGAAGATGATGGC-3′	[48]
	Reverse: 5′-TCAGCATCATCCTCATCTCG-3′	

* Gene specific primers were designed with Primer-BLAST http://www.ncbi.nlm.nih.gov/tools/primer-blast (accessed on 12 June 2021).

## Data Availability

The data that support the findings of this study are available from the corresponding author upon reasonable request.
